# Microvascular dysfunction in COVID-19: the MYSTIC study

**DOI:** 10.1007/s10456-020-09753-7

**Published:** 2020-10-14

**Authors:** Alexandros Rovas, Irina Osiaevi, Konrad Buscher, Jan Sackarnd, Phil-Robin Tepasse, Manfred Fobker, Joachim Kühn, Stephan Braune, Ulrich Göbel, Gerold Thölking, Andreas Gröschel, Hermann Pavenstädt, Hans Vink, Philipp Kümpers

**Affiliations:** 1 Department of Medicine D, Division of General Internal and Emergency Medicine, Nephrology, and Rheumatology, University Hospital Münster, Albert-Schweitzer- Campus 1, 48149 Münster, Germany; 2 Department of Cardiology and Angiology, University Hospital Münster, Albert- Schweitzer-Campus 1, 48149 Münster, Germany; 3 Department of Medicine B for Gastroenterology, Hepatology, Endocrinology, Clinical Infectiology, University Hospital Münster, Albert-Schweitzer-Campus 1, 48149 Münster, Germany; 4 Center for Laboratory Medicine, University Hospital Münster, Albert-Schweitzer- Campus 1, 48149 Münster, Germany; 5 Institute of Virology, University Hospital Münster, Albert-Schweitzer-Campus 1, 48149 Münster, Germany; 6 Departmenf of medical internal intensive care and emergency medicine, St. Franziskus-Hospital GmbH, 48145 Münster, Germany; 7 Department of Anaesthesiology and Critical Care, St. Franziskus-Hospital GmbH, 48145 Münster, Germany; 8 Department of Internal Medicine and Nephrology, University Hospital Münster Marienhospital Steinfurt, 48565 Steinfurt, Germany; 9 Department of Pulmonology, Clemenshospital, Münster, Germany; 10 Department of Physiology, Cardiovascular Research Institute Maastricht, Maastricht University, Maastricht, The Netherlands

**Keywords:** COVID-19, Sublingual microscopy, Endothelial glycocalyx, Endotheliopathy, Microcirculation

## Abstract

**Rationale:**

Pre-clinical and autopsy studies have fueled the hypothesis that a dysregulated vascular endothelium might play a central role in the pathogenesis of ARDS and multi-organ failure in COVID-19.

**Objectives:**

To comprehensively characterize and quantify microvascular alterations in patients with COVID-19.

**Methods:**

Hospitalized adult patients with moderate-to-severe or critical COVID-19 (*n* = 23) were enrolled non-consecutively in this prospective, observational, cross-sectional, multi-center study. Fifteen healthy volunteers served as controls. All participants underwent intravital microscopy by sidestream dark field imaging to quantify vascular density, red blood cell velocity (*V*_RBC_), and glycocalyx dimensions (perfused boundary region, PBR) in sublingual microvessels. Circulating levels of endothelial and glycocalyx-associated markers were measured by multiplex proximity extension assay and enzyme-linked immunosorbent assay.

**Measurements and main results:**

COVID-19 patients showed an up to 90% reduction in vascular density, almost exclusively limited to small capillaries (diameter 4–6 µm), and also significant reductions of *V*_RBC_. Especially, patients on mechanical ventilation showed severe glycocalyx damage as indicated by higher PBR values (i.e., thinner glycocalyx) and increased blood levels of shed glycocalyx constituents. Several markers of endothelial dysfunction were increased and correlated with disease severity in COVID-19. PBR (AUC 0.75, *p* = 0.01), ADAMTS13 (von Willebrand factor-cleaving protease; AUC 0.74, *p* = 0.02), and vascular endothelial growth factor A (VEGF-A; AUC 0.73, *p* = 0.04) showed the best discriminatory ability to predict 60-day in-hospital mortality.

**Conclusions:**

Our data clearly show severe alterations of the microcirculation and the endothelial glycocalyx in patients with COVID-19. Future therapeutic approaches should consider the importance of systemic vascular involvement in COVID-19.

**Electronic supplementary material:**

The online version of this article (10.1007/s10456-020-09753-7) contains supplementary material, which is available to authorized users.

## Introduction

Despite about 26 million infections and nearly 870,000 deaths worldwide (04 September 2020), the pathophysiological pathways responsible for the wide clinical spectrum of COVID-19 remain still incompletely understood [1, 2]. Although the natural course of the disease in individuals infected with the severe acute respiratory syndrome coronavirus 2 (SARS-CoV-2) is often mild, up to 15% of patients need hospital treatment [3]. These patients not only develop pulmonary disease, eventually culminating in acute respiratory distress syndrome (ARDS), but also display a myriad of extrapulmonary symptoms, including acute kidney injury (AKI), acute cardiac injury, coagulopathy, thromboembolic complications, including stroke and pulmonary embolism, and circulatory shock [2, 4]. Elegant in vitro experiments and *autopsy* studies revealed that SARS-CoV-2 binds to amply expressed angiotensin-converting enzyme 2 (ACE2) receptor and infests directly the endothelial cells [5, 6]. This finding has fueled the hypothesis that COVID-19 is actually a vascular illness and that systemic leakiness and adhesiveness of the dysregulated vascular endothelium might play a central role in the pathogenesis of ARDS and multi-organ failure [7–9].

Long underestimated as a passive lining of blood vessels, the vascular endothelium is now perceived as an independent organ system that is centrally involved in the control of thrombosis and thrombolysis, platelet and leucocyte interaction with the vessel wall, regulation of vascular tone, and the passage of fluids [10]. More recent data show that the endothelium is shielded against pathogenic insults by the endothelial glycocalyx (eGC)—a gel-like, negatively charged, up to 3-µm-thick layer consisting of highly sulfated glycosaminoglycans and proteoglycans. In bacterial sepsis, damage of the eGC plays a causative role in leukocyte recruitment, hyperpermeability, and the development of end-organ damage, especially ARDS and AKI [10].

Therefore, the aim of this prospective, cross-sectional, multi-center study was to characterize and quantify endothelial alterations in patients with moderate-to-severe or critical illness due to COVID-19. Besides multiplex analysis of circulating endothelial and glycocalyx-associated markers, we used a novel state-of-the-art image acquisition and analysis approach to detect *in vivo* even subtle alterations of the sublingual microcirculation.

## Materials and methods

### Study population and study design

This multi-center, prospective, observational, cross-sectional study took place from May to June 2020 in the intensive care units (ICU) and intermediate care wards (IMC) of the University Hospital Münster (UKM) and three local teaching hospitals (St. Franziskus-Hospital Münster, Clemens Hospital Münster, UKM-Marienhospital Steinfurt). The study was approved by the competent ethics committee (amendments of 2016-073-f-S) and was performed in accordance with the Declaration of Helsinki.

After written informed consent was obtained from the patients or their legal representatives, adult hospitalized patients with moderate/severe (IMC) or critical (ICU) COVID-19 disease were enrolled in a non-consecutive fashion by the same team of investigators. Fifteen apparently healthy volunteers served as controls.

Demographic, laboratory, and physiological variables and scores were documented for each participant immediately before the assessment of the sublingual microvasculature. Sublingual video microscopy was performed by an experienced physician. Plasma samples from patients (*n* = 23) and controls (*n* = 11) were obtained, immediately centrifuged at 4 °C with 4000×*g* for 10 min, and stored at − 80 °C for further analysis of the endothelial and inflammation components.

### Follow-up and outcome definition

Patients’ clinical course was prospectively followed up for a period of 60 days after study inclusion or until hospital discharge. In-hospital mortality was the primary outcome studied. Secondary outcomes studied were (a) development of moderate/severe ARDS during hospital stay, defined as a ratio of arterial oxygen partial pressure (PaO_2_ in mmHg) to fractional inspired oxygen (FiO_2_ expressed as a fraction) ≤ 200 mmHg based on the Berlin criteria [11], and (b) presence of thrombotic events, defined as the presence or development of clinically relevant vascular thrombotic or thromboembolic events.

### In vivo assessment of the sublingual microcirculation and glycocalyx dimensions

Details of the technique used to assess the sublingual microcirculation are provided in the online data supplement. Briefly, a sidestream dark field (SDF) camera (CapiScope HVCS, KK Technology, Honiton, UK) coupled to the GlycoCheck™ software (Microvascular Health Solutions Inc., Alpine, UT, USA) was used to visualize the sublingual microvasculature on the bedside. The software calculates the dynamic lateral movement of RBCs into the permeable part of the eGC layer, expressed as the perfused boundary region (PBR, in µm) — an inverse parameter of endothelial glycocalyx dimensions.

Vascular perfused density (mm/mm^2^) can be determined from the number of vascular segments containing RBCs multiplied by vascular segment length (10 µm). All detected RBC-containing vascular segments with a diameter between 4 and 25 µm were automatically counted in the video recordings of each subject. Perfused vascular density (hereafter vascular density) was normalized to tissue surface area. The pooled density of capillaries between 4 and 6 µm is defined as *capillary* density (*D*_4−6µm_).

RBC velocities (µm/s) are expressed in individual vessel segments by cross correlation of longitudinal RBC intensity profiles between consecutive frames of recorded videos and are calculated by dividing RBC displacement by the time between video frames. A correlation coefficient of ≥ 0.85 was required to allow accurate estimates of longitudinal RBC displacement. The median RBC velocities of the capillaries with a diameter between 4 and 7 µm were defined as capillary RBC velocity (*V*_RBC_).

To avoid contamination of the equipment, laptop, cables, and camera were covered with a transparent disposable plastic wrap. Between measurements, the whole equipment was cleaned twice with antiviral wipes appropriate for medical products. The physician conducting the video microscopy was equipped with a full personal protective equipment (PPE), as instructed, and was approved by our local department of hygiene.

### Circulating markers of endothelial dysfunction, inflammation, and injury

The endothelial markers analyzed in this study were selected a priori because of their pathophysiological relevance for the integrity of the vascular barrier and/or COVID-19 pathophysiology, respectively. Circulating levels of endothelial growth factor A and D (VEGF-A, VEGF-D), angiopoietin-1 (Angpt-1), von Willebrand factor-cleaving protease (ADAMTS13), soluble angiopoietin-1 receptor (soluble TIE2), tissue factor (TF), soluble thrombomodulin (TM), shed ectodomain of angiotensin-converting enzyme 2 receptor (ACE2), and tumor necrosis factor-alpha (TNF-α) were measured in plasma by a customized multiplex proximity extension assay (Olink, Utrecht, the Netherlands). These protein concentrations are presented in arbitrary units (arb. units) on a linear normalized scale.

Plasma levels of glycocalyx-associated proteins syndecan-1 (Diaclone, Besançon, France) and hyaluronic acid (HA; Echelon Biosciences Inc., Salt Lake City, UT, USA), as well as angiopoietin-2 (Angpt-2; R&D Systems, Minneapolis, USA) were measured using commercially available enzyme-linked immunosorbent assay (ELISA) kits according to the manufacturer’s instructions. Soluble Fms-like tyrosine kinase-1 (sFlt-1), high-sensitive troponin (hs-Troponin), d-dimer, and interleukin-6 (IL-6) were measured as part of clinical routine in the center for laboratory medicine (UKM, Münster, Germany). All measurements were performed in a blinded fashion.

### Statistical analysis

Data are presented as absolute numbers, percentages, and medians with corresponding 25th and 75th percentiles (interquartile range; IQR), as appropriate. The non-parametric Mann–Whitney *U* test and the chi-square test were used to compare parameters between groups. To correct for multiple testing in comparisons of microcirculation parameters per diameter class, we used the false discovery rate (FDR) approach of Benjamini, Krieger, and Yekutieli, setting a *q*-value < 0.05 as significant. Spearman rank correlation coefficient (rs) was used to assess correlations between variables. Receiver–operator characteristic (ROC) analysis was used to assess the area under the curve (AUC) and identify optimal cut-off values. The distribution of the time-to-event variables was estimated using the Kaplan–Meier method with log-rank testing. All the tests used were two-sided, and statistical significance was set at *p* < 0.05. SPSS version 26 (IBM Corporation, Armonk, NY, USA) and GraphPad Prism version 8.4.3 (GraphPad Prism Software Inc., San Diego, CA, USA) were used for statistical analyses and preparation of figures.

## Results

The COVID-19 cohort consisted of 23 adult patients with median (IQR) age of 62 (54–75) years. Most of the patients were male (*n* = 20; 87%), overweight (BMI: 26.6 kg/m^2^ [IQR: 23.4–29.4]), and had a history of arterial hypertension (*n* = 15; 65%). Fourteen patients (61%) were mechanically ventilated at study inclusion. The clinical and demographic characteristics of COVID-19 patients are shown in Table 1. Fifteen apparently healthy controls (age [IQR]: 35 [28–66]; 53% female) determined the normal range of the different markers.

**Table 1:** Baseline characteristics of COVID-19 patients stratified for mechanical ventilation

Variable	All patients	w/o Mechanical ventilation	w/ Mechanical ventilation	*p* value^#^
Number of participants (*n*; %)	23	9 (39.1)	14 (60.9)	–
Female sex (*n*; %)	3 (13)	2 (22)	1 (7)	0.30
Age (years, median (IQR))	62 (54–75)	64 (53–77)	61 (55–67)	0.52
BMI (kg/m^2^, median (IQR))	26.6 (23.4–29.4)	24.3 (22.9–27.9)	27.5 (24.1–32.6)	0.11
Positive nasopharyngeal swab (*n*; %)	22 (96.7)	9 (100)	13 (92.9)*	> 0.99
Viraemia at study inclusion (*n*; %)	2 (8.7)	0 (0)	2 (14.3)	0.50
Days from hospital admission (median (IQR))	7 (1–17)	2 (0.5–21.5)	10 (2.5–18.3)	0.48
ICU at study inclusion (*n*; %)	16 (69.5)	2 (22.2)	14 (100)	0.0001
SOFA score (pts, median (IQR))	6 (2–13)	2 (0–4)	11 (6–16)	< 0.0001
SOFA respiratory (pts, median (IQR))	2 (1–3)	0 (0–2.5)	2.5 (2–3)	0.02
PaO_2_/FiO_2_ ratio (median (IQR))	222.50 (164.17–339.29)	342.85 (196.32–502.38)	194.88 (145.76–234.0)	0.02
MAP (mmHg, median (IQR))	78.0 (68.7–89.7)	85.3 (77.3–96.5)	73.2 (64.4–85.7)	0.01
Vasopressors (*n*; %)	6 (26.1)	1 (11.1)	5 (35.7)	0.34
Norepinephrine Dose (µg/kg/min, median (IQR))	0 (0–0.03)	0	0 (0.0–0.5)	0.34
Acute dialysis (*n*; %)	7 (30.4)	0	7 (50)	0.02
60-days in-hospital mortality (*n*; %)	6 (26.1)	1 (11.1)	5 (35.7)	0.34
CCI score (pts, median (IQR))	1 (0–3)	1 (0–2)	0 (0–3.25)	0.73
Comorbidities (*n*; %)				
Arterial hypertension	15 (65.2)	4 (44.4)	11 (78.6)	0.18
Chronic respiratory disease	4 (17.4)	2 (22.2)	2 (14.2)	> 0.99
Congestive heart failure	6 (26.1)	2 (22.2)	4 (28.6)	> 0.99
Rheumatologic disease	4 (17.4)	2 (22.2)	2 (14.2)	> 0.99
Malignancy	5 (21.7)	3 (33.3)	2 (14.3)	0.34
Diabetes mellitus	0	0	0	–
Sublingual microscopy (median (IQR))				
PBR (µm)	2.30 (2.10–2.51)	2.16 (2.01–2.24)	2.44 (2.28–2.55)	0.002
* D*_4−6 µm_ (mm/mm^2^)	24.92 (14.07–52.19)	33.63 (18.22–66.08)	20.80 (11.46–46.79)	0.11
* V*_RBC_ (µm/s)	90.0 (79.9–106.8)	106.4 (95.5–121.0)	81.7 (74.3–91.9)	0.004
Endothelial markers (median (IQR))				
Syndecan-1 (ng/ml)	163.6 (33.5–246.5)	29.9 (22.8–82.4)	239.0 (162.8–251.5)	< 0.0001
Hyaluronic acid (ng/ml)	234.2 (139.8–487.6)	139.9 (113.9–393.9)	240.6 (177.8–723.7)	0.20
Angpt-1 (arb. unit)	122.1 (53.7–183.3)	141.5 (105.0–231.5)	95.7 (40.5–172.4)	0.16
Angpt-2 (ng/ml)	5.5 (3.9–6.6)	4.2 (2.3–5.5)	6.4 (5.0–6.7)	0.06
Soluble Tie2 (arb. unit)	122.0 (98.4–142.2)	104.9 (94.9–135.1)	135.0 (101.1–156.3)	0.16
VEGF-A (arb. unit)	2659.6 (2342.1–4430.5)	2442.6 (1717.2–2660.7)	4067.2 (2584.4–4883.3)	0.005
VEGF-D (arb. unit)	107.4 (80.8–141.3)	140.3 (102.2–147.6)	91.8 (62.3–130.3)	0.051
sFLT-1 (pg/ml)	144.0 (86.1–575.0)	86.1 (71.3–116.5)	471.5 (131.7–672.3)	0.003
ADAMTS13 (arb. unit)	19.0 (16.9–20.7)	20.2 (19.9–24.3)	17.3 (14.7–18.8)	0.001
ACE2 (arb. unit)	27.6 (10.4–41.6)	10.6 (6.4–26.7)	34.1 (23.5–51.8)	0.007
Soluble thrombomodulin (arb. Unit)	685.2 (536.3–909.1)	537.3 (416.0–649.3)	857.1 (646.6–1036.6)	0.004
Tissue factor (arb. Unit)	53.8 (41.0–68.4)	50.85 (30.4–63.4)	59.0 (46.5–69.9)	0.21
d-Dimers (mg/l)	2.52 (1.70–6.04)	1.70 (0.72–2.31)	4.83 (2.90–10.30)	0.001
Inflammatory markers (median (IQR))				
CRP (mg/dl)	12.2 (4.5–21.9)	10.3 (0.9–16.5)	14.2 (11.1–28.0)	0.06
IL-6 (pg/ml)	62.0 (25.0–131.0)	30.0 (9.5–154.0)	69.0 (48.5–154.3)	0.14
Ferritin (µg/l)	1085 (468–1499)	712 (301–972)	1244 (582–2079)	0.12
PCT (ng/ml)	0.64 (0.13–3.16)	0.13 (0.11–0.43)	2.10 (0.40–5.60)	0.006
TNF-a (arb. unit)	8.6 (6.1–10.8)	6.9 (4.8–7.8)	10.3 (8.2–13.3)	0.007
hs-Troponin (ng/l)	76.7 (27.2–198.0)	28.8 (10.5–65.4)	121.0 (36.6–251.0)	0.06

^#^*p*-values were calculated between the two COVID-19 groups (with and without mechanical ventilation)

*One patient had negative nasopharyngeal swab, but typical signs, symptoms, and radiological findings on the chest computed tomography (CT) at study inclusion

Abbreviations:

*ACE2* shed ectodomain of angiotensin-converting enzyme 2 receptor, *ADAMTS13* a disintegrin and metalloprotease with thrombospondin type 1 motif member 13, *Angpt-1* Angiopoietin-1, *Angpt-2* Angiopoietin-2, *Arb. unit* Arbitrary unit, *BMI* Body mass index, *CCI score* Charlson Comorbidity Index, *CRP* C-reactive protein, *hs-Troponin* high-sensitive Troponin, *ICU* Intensive care unit, *IL-6* Interleukin-6, *IQR* interquartile range, *MAP* Mean arterial pressure, *PBR* Perfused boundary region, *PCT* Procalcitonin, *RBC* Red blood cell, *SOFA score* Sequential Organ Failure Assessment score, *Soluble Flt-1* Soluble Fms-like tyrosine kinase-1, *Soluble Tie2* Angiopoietin-1 receptor, *TNF-a* Tumor necrosis factor a, *VEGF-A* Vascular endothelial growth factor A, *VEGF-D* Vascular endothelial growth factor D, *w/* with, *w/o* without

### Vascular density, RBC velocity, and glycocalyx damage

**Fig. 1:**
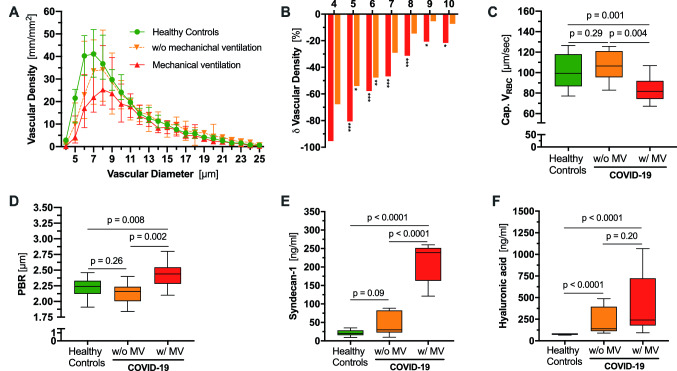
Endothelial glycocalyx dimensions in vivo and in vitro and capillary density in COVID-19 patients with (w/) and without (w/o) mechanical ventilation (MV) and healthy controls. **a** Median and IQR values of vascular density of healthy controls and COVID-19 patients based on the diameter class from 4 to 25 µm. **b** Bar charts showing the percentage of loss of vascular density in COVID-19 patients with (red) and without (orange) mechanical ventilation compared to healthy controls (diameter class from 4 to 10 µm). *q < 0.05, **q < 0.01, ***q < 0.001 Boxplots of **c** of capillary *V*_RBC_, **d** PBR values, and endothelial glycocalyx constituents **e** syndecan-1 and **f** hyaluronic acid of healthy controls (green) and COVID-19 patients with (red) or without (orange) mechanical ventilation (MV) *p < 0.05, **p < 0.01, ***p < 0.001

Sublingual video microscopy showed severe alterations of the microvasculature in COVID-19 patients. First, we compared vascular density between healthy controls and COVID-19 patients in a diameter-class-wise fashion (Fig. 1a, b). This approach revealed a tremendous decrease in vascular density in the diameter classes 4–10 µm in mechanically ventilated patients compared to controls. A similar, albeit less pronounced, pattern was observed in patients without need for mechanical ventilation (MV) (Fig. 1a, b). Interestingly, the capillary density loss (*D*_4−6µm_) in COVID-19 patients correlated with d-dimer levels (rs = − 0.43, *p* = 0.04, Figure E1: Online Supplement). Density of microvessels > 10 µm was not different between the three groups, indicating that the small capillaries are primarily affected. Moreover, *V*_RBC_ in these capillaries was significantly lower in mechanically ventilated patients compared to healthy individuals (81.7 µm/s [74.3–91.9] vs. 106.4 µm/s [95.5–121.0], *p* = 0.004). COVID-19 patients without need for MV showed normal *V*_RBC_ values (106.4 µm/s [95.5–121.1] vs. 99.4 µm/s [86.8–118.0], *p* = 0.29) (Fig. 1c).

**Fig. 2:**
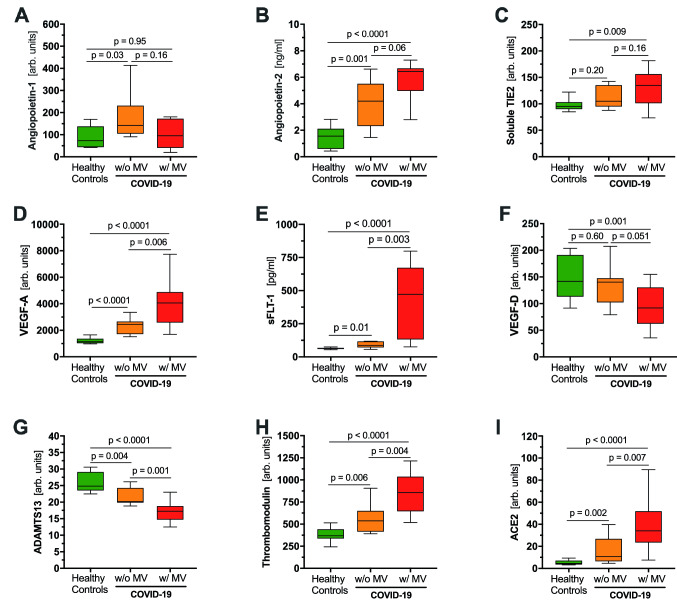
Comparisons of a priori selected markers of endothelial dysfunction in COVID-19 patients with and without mechanical ventilation and healthy controls. Boxplots of **a** angiopoietin-1, **b** angiopoietin-2, **c** soluble TIE2, **d** VEGF-A, **e** sFLT-1, **f** VEGF-D, **g** ADAMTS13, **h** soluble thrombomodulin, and **i** ACE2 between healthy controls and COVID-19 patients with (red) and without (orange) mechanical ventilation (MV)

Next, we analyzed the PBR which has been identified as a robust and reliable estimate of glycocalyx damage [12–14]. COVID-19 patients on MV showed significantly higher PBR values (i.e., thinner glycocalyx layer) compared to non-ventilated patients (2.44 µm [2.28–2.55] vs. 2.16 µm [2.01–2.24], *p* = 0.002) and controls (2.44 µm [2.28–2.55] vs. 2.24 [2.12–2.33], *p* = 0.008), respectively. This finding was corroborated by highly elevated plasma levels of HA and syndecan-1, circulating markers of glycocalyx shedding, which markedly increased with need for MV in COVID-19 patients (Fig. 1d–f). PBR values and syndecan-1 concentrations correlated moderately not only with each other (rs = 0.50 [95% CI 0.10–0.76], *p* = 0.02), but also with further markers of endothelial dysfunction and injury (Figure E1: Online Supplement). In summary, COVID-19 patients show severe alterations of the microcirculation and the endothelial glycocalyx, which increase with disease severity.

### Circulating markers of endothelial dysfunction, inflammation, and injury

The endothelium-specific Angpt/Tie2system controls endothelial activation in a non-redundant fashion [15]. While the protective TIE2-agonisic ligand Angpt-1 was not different between the groups, its leakage-inducing antagonist Angpt-2 was significantly increased in COVID-19 patients, especially in those on MV. Soluble TIE2, generated by proteolytic cleavage and release of the ectodomain from full-length receptor located at the cell surface [16], increased in COVID-19 patients (Table 1; Fig. 2a–c).

**Fig. 3:**
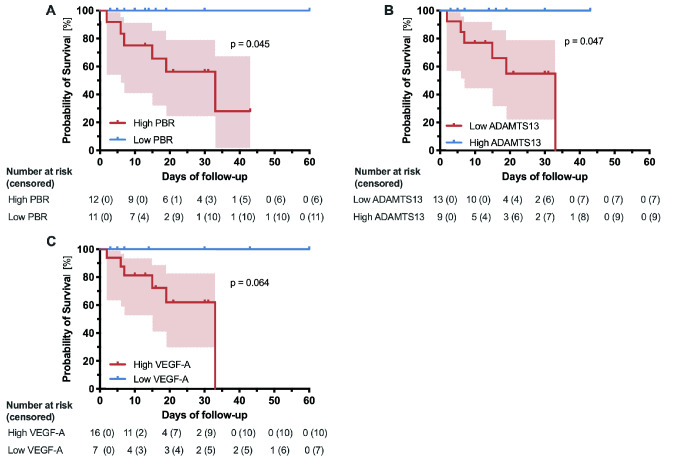
Survival probability of COVID-19 patients according to different endothelial markers. Kaplan–Meier curves with 95% CIs showing survival probability of COVID-19 patients with **a** low/high PBR, **b** low/high ADAMTS13, and **c** low/high VEGF-A. ^#^ADAMTS13 of one patient could not be measured due to technical reasons

The vasodilating and permeability factor VEGF-A and its endogenous inhibitor sFlt-1, a truncated and circulating form of the VEGF-A receptor Flt-1, were markedly increased in COVID-19 patients and correlated with disease severity. VEGF-D, which promotes angiogenesis and lymphangiogenesis [17], was lower in COVID-19 patients on MV compared to healthy controls and patients without need for MV (Table 1; Fig. 2d–f).

Levels of ADAMTS13, an antithrombotic metalloprotease which cleaves highly adhesive large von Willebrand factor (VWF) multimers after their release from activated endothelium [18], decreased significantly with increasing COVID-19 severity (Fig. 2g). Circulating fragments of TM, an endothelial injury marker, were markedly increased in COVID-19 patients and correlated with disease severity (Fig. 2h). Levels of the shed ectodomain of ACE2, the main host cell receptor of SARS-CoV-2, correlated with disease activity and increased approximately tenfold in COVID-19 patients on MV (Fig. 2i).

Among routine markers of inflammation, PCT and TNF-α, but not CRP, IL-6 or ferritin, were higher in COVID-19 patients on MV compared to those not on MV (Table 1).

Figure E1 (online supplement) shows a correlation matrix including *D*_4−6µm_, *V*_RBC_, glycocalyx and endothelial-associated markers, and inflammatory cytokines. Of note, markers of glycocalyx damage, namely PBR and syndecan-1, correlated positively with d-Dimer levels (PBR: rs = 0.56, *p* < 0.01; syndecan-1: rs = 0.56; *p* < 0.01 ), ACE2 (PBR: rs = 0.46, *p* < 0.05; syndecan-1: rs = 0.59; *p* < 0.01), and inversely with ADAMTS13 antigen levels (PBR: rs = − 0.52, *p* < 0.05; syndecan-1: rs = − 0.64, *p* < 0.01). *D*_4−6µm_ correlated inversely with d-dimer (rs = − 0.43, *p* < 0.05) and ACE2 (rs = − 0.46, *p* < 0.05) antigen levels. ADAMTS13 showed moderate to strong inverse association with Angpt-2 (rs = − 0.47, *p* < 0.05), ACE2 (rs = − 0.57, *p* < 0.01), TM (rs = − 0.64, *p* < 0.01), d-Dimers (rs = − 0.52, *p* < 0.05), and VEGF-A (rs = − 0.85, *p* < 0.001).

In summary, our data show a plausible but complex pattern of endothelial dysfunction and damage, in which counter-regulatory mechanisms seem to be operative.

### Association of endothelial dysfunction with clinical parameters and outcome

Table E1 (Online Supplement) shows correlations of selected endothelial parameters with metric clinical variables, such as PaO_2_/FiO_2_ index, Sequential organ failure assessment (SOFA) score, Sepsis-induced coagulopathy (SIC) score, hs-troponin, and hemodynamics. The performance of endothelial markers to predict clinical outcomes was evaluated using ROC analysis (Table E2 – online supplement). The AUC of ADAMTS13 and syndecan-1 was 0.91 (*p* < 0.0001) while ACE2, d-Dimer, PBR, and VEGF-A showed AUCs ≥ 0.85 (*p* < 0.0001) in predicting development of moderate-to-severe ARDS during hospital stay. d-Dimers showed the highest AUC for association of thrombotic events (AUC 0.80, *p* = 0.001), followed by markers of glycocalyx damage, PBR (AUC 0.78, *p* = 0.02), and syndecan-1 (AUC 0.76, *p* = 0.02).

Regarding in-hospital 60-day mortality, PBR (AUC 0.75, *p* = 0.01), ADAMTS13 (AUC 0.74, *p* = 0.02), and VEGF-A (AUC 0.73, *p* = 0.04) showed the best discriminatory ability. ROC-derived cut-off values of these markers were used to divide the COVID-19 cohort into two groups of high and low values, respectively. As shown by Kaplan–Meier curves, high PBR values (*p* = 0.045) and reduced ADAMTS13 antigen levels (*p* = 0.047) were associated with mortality (Fig. 3).

**Fig. 4:**
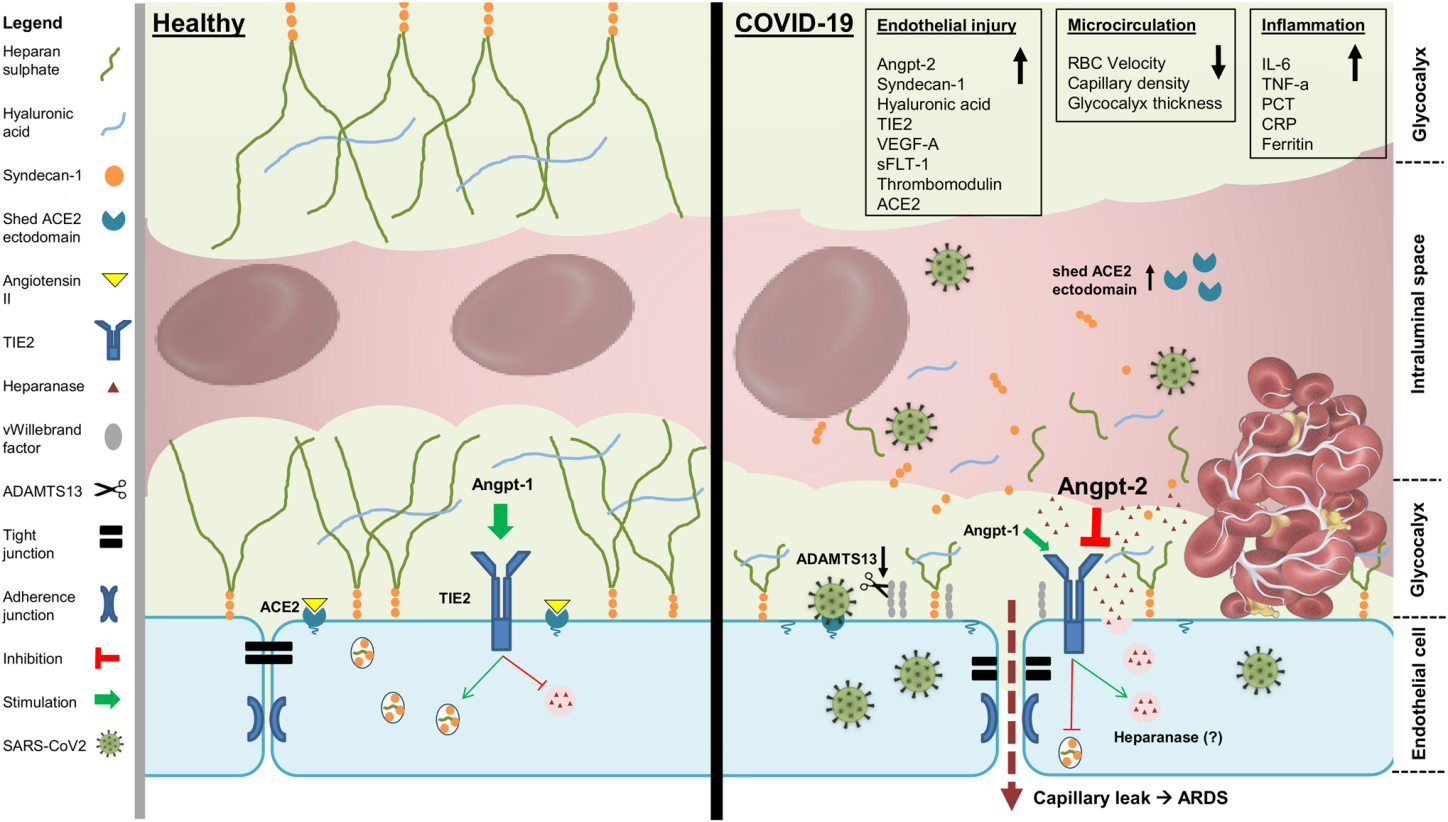
Endothelial, glycocalyx, and microcirculation damage in COVID-19. The image illustrates a simplified overview summarizing the findings of this study

## Discussion

Our comprehensive analysis approach, comprising functional and biomarker data clearly shows severe alterations of the microcirculation and the endothelial glycocalyx in patients with COVID-19. Several of those markers were closely related to disease severity and predicted ARDS development. Of note, sublingual glycocalyx thickness and circulating ADAMTS13 and VEGF-A levels, but not initially proposed (inflammatory) markers such as ferritin, CRP, IL-6 or hs-troponin, predicted 60-day in-hospital mortality. Thus, our data provide further evidence for the importance of systemic vascular involvement in COVID-19.

An unspecified, small reduction in total and perfused vascular density in sublingual microvessels of ventilated COVID-19 patients has been recently reported [19, 20]. However, neither outcome nor endothelial or glycocalyx markers have been assessed in these studies. Our detailed per-diameter analysis shows for the first time the exact localization (i.e., *true* capillaries, D_4 − 6µm_) and incredible extent of capillary dropout (> 60%) in mechanically ventilated COVID-19 patients. Capillary impairment (V_RBC_ and D_4 − 6 µm_) correlated with SOFA and SIC score as well as oxygenation index, indicating that sublingual capillaries are, at least in part, representative of the pulmonary ones. Capillary clogging by fibrinous microthrombi, which has been shown by autopsy studies in lungs from COVID-19 patients [5, 21], is thus the putative histopathological correlate of capillary rarefication in *sublingual* capillaries as well. Consistent with this notion, d-dimers levels were closely associated with microcirculatory alterations in our and the abovementioned studies [19, 20].

Local formation of microthrombi and subsequent capillary clogging requires a switch of the endothelial phenotype from quiescence to a pro-adhesive, pro-inflammatory activational state. Interestingly, this process is non-redundantly controlled by Tie2, a receptor that is highly enriched in the endothelium and actively signals vascular quiescence [22]. Under physiological conditions, Tie2 is tonically activated by Angpt-1, a vasculoprotective protein secreted by peri-endothelial cells and platelets [23]. In human sepsis, its intrinsic antagonist called Angpt-2 is rapidly released from activated endothelium, competitively inhibits Tie2 and predicts mortality as a biomarker [15, 24, 25]. Elegant translation work by Higgins et al. showed that Angpt-2-driven Tie2 deactivation is central to microvascular thrombus formation in sepsis [26]. Angpt-2 was already increased in non-ventilated SARS-CoV-2 infected patients, indicating that Angpt-2 may unleash endothelial inflammation in COVID-19 early on. In this regard, Smadja et al. identified an Angpt-2 cut-off of 5.0 ng/ml as best early predictor for ICU admission in 40 consecutive COVID-19 patients admitted to the emergency department [27]. Although Angpt-2 correlated with SOFA and SIC scores, it didn´t predict clinical outcomes in our cohort of COVID-19 patients.

Among the VEGF system, VEGF-D [28], and sFLT-1 [29] have been discussed in the pathophysiology of COVID-19. Kong et al. reported a *positive* correlation between VEGF-D: a still not fully understood member of the VEGF family – and disease severity in COVID-19 patients [28]. Surprisingly, we found a clear *negative* correlation between VEGF-D and SOFA score. A reason for this discrepancy could be the cross-sectional design of our study, as VEGF-D levels seem to fluctuate during hospital stay [28]. However, VEGF-A, a strong permeability-inducing factor related to disease severity and survival in sepsis [30], correlated positively with disease severity and ARDS development in our cohort. The bioavailability of elevated VEGF-A, however, is difficult to deduce, since sFlt-1 acts as a scavenger receptor and neutralizes VEGF-A. Under healthy conditions, sFlt-1 binds electrostatically to proteoglycans and is, thus, buffered within the glycocalyx [31]. Not surprisingly, glycocalyx damage coincides with excess sFlt-1 levels in COVID-19 patients.

In a comprehensive study on the hypercoagulability in COVID-19, Goshua et al. found evidence of excessive VWF release, but absence of DIC. Both VWF antigen and soluble thrombomodulin, a specific marker of endothelial cell injury were significantly correlated with mortality [32]. In sepsis, elevated VWF antigen and activity can be accompanied by reductions in the ADAMTS13 metalloproteinase responsible for cleaving ultra-large VWF multimers into smaller VWF forms. Mounting VWF/ADAMTS13 imbalance, culminating in the accumulation of uncleaved VWF molecules has been shown to increases the risk of developing secondary thrombotic microangiopathy in sepsis. In line with this finding, ADAMTS13 antigen correlated inversely with glycocalyx damage and SOFA score and predicted ARDS and 60-day mortality in our study [33]. Evidence of reduced ADAMTS13 activity in the presence of schistocytes (~ 2–4%) appeared compatible with thrombotic microangiopathy (TMA) secondary to COVID-19 [34].

Although some endothelial markers, such as thrombomodulin, have been already linked to mortality in COVID-19 patients [32], the predictive value of the PBR as an estimate of glycocalyx thickness has not been reported yet. Interestingly, previous work by our group revealed that damage and refurbishment of the eGC are tightly controlled by Tie2 in human sepsis [12, 35]. Mechanistically, Angpt-2 causes heparanase secretion from distinctive cellular storage pools with consecutive enzymatic degradation of the glycocalyx [35]. A groundbreaking translational study by Schmidt et al. provided compelling evidence that prevention of heparanase-mediated degradation of the pulmonary endothelial glycocalyx—by blocking heparanase via a non-coagulant heparin fragment—is sufficient to eliminate vascular hyperpermeability and ARDS in murine endotoxemia [36]. It is intriguing to speculate that a possible off-target effect of heparin, which appears to improve mortality in COVID-19 patients [37], may be the stabilization or restoration of the endothelial glycocalyx. However, the involvement of heparanase in COVID-19-induced glycocalyx damage has yet to be formally demonstrated and needs further study.

We acknowledge some limitations in our study. First, sublingual video microscopy in COVID-19 patients is challenging due to the increased risk of transmission. We therefore started the measurements only after we had developed a safe hygiene protocol. Given that the number of infections in the City of Münster was very low anyway, we had to conduct the study in a cross-sectional fashion with a limited sample size. Therefore, we cannot exclude that the identified predictors may be less meaningful in early disease stages or outpatients, respectively. Second, this study was neither designed nor powered to test the performance of endothelial or microvascular parameters for outcome prediction. However, our findings are plausible, hypothesis-generating, and clearly deserve validation in larger, prospective studies with serial blood drawings and microscopic measurements. Third, although participating centers regularly exchanged information on the basic treatment strategies, these may have differed in detail and, therefore, could have influenced the results. Lastly, most of the COVID-19 patients were of male sex. Indeed, male individuals are more often infected by SARS-CoV2. Our results are therefore only transferable to the female sex with caution.

## Conclusion and outlook

Our data clearly support that COVID-19 is accompanied by endothelial activation, glycocalyx damage, and severe capillary impairment (Fig. 4). Although our data cannot prove causality, it is very likely that COVID-19 has a distinctive vascular phenotype or even represents a novel vascular multisystemic disease. A precise quantification of endothelial glycocalyx damage could be a new parameter for outcome prediction. Future prediction models and therapeutic approaches should, thus, consider the importance of the vascular endothelium and its glycocalyx in COVID-19.

## Electronic supplementary material

Below is the link to the electronic supplementary material.

**Fig. E1 – Online supplement** Correlation matrix (Spearman) with markers of microvascular dysfunction, endothelial activation, glycocalyx breakdown, and coagulopathy for all COVID-19 patients. Statistical significance is marked as following: * *p* < 0.05, ** *p* < 0.01, *** *p* < 0.001. ACE2 = shed ectodomain of angiotensin-converting enzyme 2 receptor; ADAMTS13 = a disintegrin and metalloprotease with a thrombospondin type 1 motif, member 13; Angpt-1 = Angiopoietin-1; Angpt-2 = Angiopoietin-2; D_4 − 6µm_ = Capillary density; PBR = Perfused boundary region; sFLT-1 = Soluble Fms-like tyrosine kinase-1; sTie2 = Angiopoietin-1 receptor; TF = Tissue factor; TM = Thrombomodulin; VEGF-A = Vascular endothelial growth factor A; VEGF-D = Vascular endothelial growth factor D; V_RBC_ = Capillary red blood cell velocity. Electronic supplementary material 1 (EPS 190 kb)

Electronic supplementary material 2 (DOCX 55 kb)

Electronic supplementary material 3 (DOCX 29 kb)

## Data Availability

The datasets used and/or analyzed during the current study are available from the corresponding author on reasonable request.

## Abbreviations

ACE2: Shed ectodomain of angiotensin-converting enzyme 2 receptor
ADAMTS13: A disintegrin and metalloprotease with a thrombospondin type 1 motif, member 13
AKI: Acute kidney injury
Angpt-1: Angiopoietin-1
Angpt-2: Angiopoietin-2
Arb. unit: Arbitrary unit
ARDS: Acute respiratory distress syndrome
AUC: Area under the curve
BMI: Body mass index
CCI score: Charlson comorbidity index
COVID-19: Corona virus disease 2019
CRP: C-reactive protein
*D*_4–6 µm_: Capillary density
eGC: Endothelial glycocalyx
FDR: False discovery rate
HA: Hyaluronic acid
hs-Troponin: High-sensitive troponin
ICU: Intensive care unit
IL-6: Interleukin-6
IMC: Intermediate care ward
IQR: Interquartile range
MAP: Mean arterial pressure
MV: Mechanical ventilation
PaO_2_/FiO_2_ index: The ratio of partial pressure of oxygen in blood (PaO_2_), in millimeters of mercury, and the fraction of oxygen in the inhaled air (FiO_2_)
PBR: Perfused boundary region
PCT: Procalcitonin
RBC: Red blood cell
RBCW: Red blood cell width
ROC: Receiver operating curve
SARS-CoV-2: Severe acute respiratory syndrome coronavirus 2
SDF: Sidestream dark field
SIC score: Sepsis-induced coagulopathy score
SOFA score: Sequential Organ Failure Assessment score
Soluble Flt-1: Soluble Fms-like tyrosine kinase-1
Soluble Tie2: Angiopoietin-1 receptor
TF: Tissue factor
TM: Thrombomodulin
TMA: Thrombotic microangiopathy
TNF-α: Tumor necrosis factor a
VEGF-A: Vascular endothelial growth factor A
VEGF-D: Vascular endothelial growth factor D
V_RBC_: Capillary red blood cell velocity
VWF: von Willebrand factor
UKM: University Hospital Münster
w/: With
w/o: Without
