# Supplementary material for: Microvascular dysfunction in COVID-19: the MYSTIC study
Source: Angiogenesis. 2020 Oct 14;24(1):145–57. doi: 10.1007/s10456-020-09753-7 (PMC7556767; doi:10.1007/s10456-020-09753-7)
Supplement: Supplementary file 2 — Electronic supplementary material 2 (DOCX 55 kb) [file 10456_2020_9753_MOESM2_ESM.docx]

**Microvascular dysfunction in COVID-19 – the MYSTIC study**

Alexandros Rovas^*^, Irina Osiaevi^*^, Konrad Buscher, Jan Sackarnd, Phil-Robin Tepasse, Manfred Fobker, Joachim Kühn, Stephan Braune, Ulrich Göbel, Gerold Thölking, Andreas Gröschel, Hermann Pavenstädt, Hans Vink, Philipp Kümpers

* contributed equally and are both considered first authors

**Corresponding author**:

Philipp Kümpers MD, Department of Medicine D, Division of General Internal Medicine, Nephrology, and Rheumatology, University Hospital Münster, Albert-Schweitzer-Campus 1, 48149 Münster, Germany

Email: philipp.kuempers@ukmuenster.de

Phone: 0049-251-83-47516

# Online Data Supplement

**Table E1: Correlations (Spearman) between markers of microvascular dysfunction and clinical parameters.**

* p<0.05, ** p<0.01, *** p<0.001

|  | **SOFA** | **MAP** | **SIC** | **PaO2/FiO2** | **hs-Troponin** | **CRP** | **PCT** | **IL-6** | **Ferritin** | **TNF-a** |
| --- | --- | --- | --- | --- | --- | --- | --- | --- | --- | --- |
| **PBR** | 0.25 | -0.22 | 0.18 | -0.08 | 0.54 *** | 0.16 | 0.17 | 0.33 | -0.002 | 0.08 |
| **D_4-6µm_** | -0.53 ** | 0.25 | -0.50 * | 0.46 * | -0.15 | -0.48 * | -0.54 * | -0.31 | -0.41 | -0.02 |
| **V_RBC_** | -0.61 ** | 0.24 | -0.41 | 0.52 * | -0.32 | -0.32 | -0.53 * | -0.44 * | -0.16 | -0.38 |
| **Syndecan-1** | 0.67 *** | -0.69 *** | 0.51 * | -0.54 ** | 0.46 * | 0.42 | 0.57 ^**^ | 0.34 | 0.35 | 0.32 |
| **Angpt-2** | 0.61 ** | -0.30 | 0.57 ** | -0.34 | 0.32 | 0.64 ** | 0.58 ** | 0.68 *** | 0.55 ** | 0.28 |
| **VEGF-A** | 0.67 ** | -0.47 * | 0.65 ** | -0.42 * | 0.44 * | 0.43 * | 0.67 ** | 0.18 | 0.24 | 0.33 |
| **VEGF-D** | -0.68 ** | 0.43 * | -0.33 | 0.41 | -0.34 | -0.33 | -0.46 * | -0.13 | -0.17 | -0.45 * |
| **sFLT-1** | 0.58 ** | -0.77 *** | 0.36 | -0.28 | 0.43 * | 0.06 | 0.32 | 0.20 | 0.12 | 0.19 |
| **ADAMTS13** | -0.72 *** | 0.52 * | -0.62 ** | 0.47 * | -0.51 * | -0.43 * | -0.71 *** | -0.28 | -0.42 * | -0.29 |
| **ACE2** | 0.50 * | -0.32 | 0.49 * | -0.69 *** | 0.37 | 0.24 | 0.45 * | 0.54 * | 0.26 | 0.15 |
| **Soluble TM** | 0.67 ** | -0.44 * | 0.50 * | -0.37 | 0.52 * | 0.16 | 0.46 * | 0.18 | 0.12 | 0.45 * |
| **D-Dimer** | 0.58 ** | -0.28 | 0.59 ** | -0.35 | 0.33 | 0.49 * | 0.38 | 0.57 ** | 0.27 | 0.25 |

Abbreviations:

ACE2 = shed ectodomain of angiotensin-converting enzyme 2 receptor, ADAMTS13 = a disintegrin and metalloprotease with thrombospondin type 1 motif member 13, Angpt-2 = Angiopoietin-2, CRP = C-reactive protein, D_4-6µm_ = Capillary density, hs-Troponin = high sensitive Troponin, IL-6 = Interleukin-6, MAP = Mean arterial pressure, PaO2/FiO = ratio of partial pressure of oxygen in blood (PaO2), in millimeters of mercury, and the fraction of oxygen in the inhaled air (FiO2) – Horowitz index, PBR = Perfused boundary region, PCT = Procalcitonin, SIC = Sepsis induced coagulopathy score, SOFA = sequential organ failure assessment score, sFlt-1 = Soluble Fms-like thyrosinkinase-1, Soluble TM = Thrombomodulin, TNF-a = Tumor necrosis factor, VEGF-A = Vascular endothelial growth factor A, VEGF-D = Vascular endothelial growth factor D, V_RBC_ = Capillary red blood cell velocity

**Table E2: ROC analysis regarding (A) development of moderate/severe ARDS during hospital stay, (B) presence of thrombotic events, (C) 60-day in-hospital mortality.**

|  | 1. **Moderate-severe ARDS** | | 1. **Thrombotic events** | | |
| --- | --- | --- | --- | --- | --- |
| **Variable** | **AUC (95% CI)** | ***P value*** | **Variable** | **AUC (95% CI)** | ***P value*** |
| ADAMTS13 | 0.91 (0.78-1.04) | <0.0001 | D-Dimer | 0.80 (0.52-0.98) | 0.001 |
| Syndecan-1 | 0.91 (0.78-1.04) | <0.0001 | PBR | 0.78 (0.55-1.0) | 0.02 |
| ACE2 | 0.90 (0.77-1.03) | <0.0001 | Syndecan-1 | 0.76 (0.54-0.97) | 0.02 |
| D-Dimer | 0.89 (0.75-1.00) | <0.0001 | ACE2 | 0.69 (0.48-0.91) | 0.08 |
| PBR | 0.88 (0.73-1.03) | <0.0001 | sFLT-1 | 0.69 (0.42-0.95) | 0.17 |
| V_RBC_ | 0.87 (0.71-1.02) | <0.0001 | Soluble TM | 0.68 (0.43-0.93) | 0.16 |
| VEGF-A | 0.85 (0.69-1.01) | <0.0001 | VEGF-D | 0.67 (0.42-0.92) | 0.18 |
| sFLT-1 | 0.83 (0.66-0.99) | <0.0001 | Soluble TIE2 | 0.64 (0.40-0.87) | 0.25 |
| TNF-a | 0.80 (0.62-0.99) | 0.002 | IL-6 | 0.63 (0.36-0.90) | 0.33 |
| PCT | 0.80 (0.61-0.99) | 0.002 | hs-Troponin | 0.62 (0.36-0.90) | 0.37 |
| Soluble TM | 0.78 (0.58-0.98) | 0.007 | V_RBC_ | 0.62 (0.34-0.89) | 0.40 |
| D_4-6µm_ | 0.75 (0.55-0.96) | 0.02 | Angpt-2 | 0.53 (0.27-0.79) | 0.83 |
| BMI | 0.75 (0.53-0.96) | 0.03 | TF | 0.53 (0.22-0.84) | 0.85 |
| hs-Troponin | 0.74 (0.50-0.98) | 0.051 | TNF-a | 0.57 (0.28-0.85) | 0.64 |
| VEGF-D | 0.71 (0.48-0.93) | 0.07 | Ferritin | 0.53 (0.25-0.81) | 0.84 |
| Angpt-2 | 0.71 (0.47-0.95) | 0.09 | Age | 0.51 (0.22-0.79) | 0.97 |
| Soluble TIE2 | 0.68 (0.44-0.91) | 0.14 | D_4-6µm_ | 0.46 (0.17-0.75) | 0.79 |
| Ferritin | 0.68 (0.44-0.91) | 0.15 | Angpt-1 | 0.45 (0.14-0.76) | 0.74 |
| IL-6 | 0.68 (0.41-0.94) | 0.19 | ADAMTS13 | 0.45 (0.20-0.66) | 0.68 |
| Angpt-1 | 0.67 (0.44-0.90) | 0.14 | HA | 0.43 (0.18-0.69) | 0.60 |
| HA | 0.63 (0.36-0.89) | 0.35 | PCT | 0.43 (0.13-0.72) | 0.62 |
| TF | 0.61 (0.35-0.87) | 0.42 | BMI | 0.42 (0.16-0.68) | 0.55 |
| Age | 0.44 (0.16-0.72) | 0.68 | VEGF-A | 0.42 (0.17-0.68) | 0.55 |
| Sex | 0.41 (0.15-0.67) | 0.49 | Sex | 0.41 (0.16-0.66) | 0.49 |

| **(C) 60-day in-hospital mortality** | | |
| --- | --- | --- |
| **Variable** | **AUC (95% CI)** | ***P value*** |
| PBR | 0.75 (0.55-0.95) | 0.01 |
| ADAMTS13 | 0.74 (0.53-0.95) | 0.02 |
| VEGF-A | 0.73 (0.51-0.94) | 0.04 |
| Age | 0.74 (0.47-1.00) | 0.08 |
| PCT | 0.70 (0.46-0.94) | 0.10 |
| Soluble TM | 0.69 (0.46-0.91) | 0.10 |
| ACE2 | 0.68 (0.43-0.93) | 0.16 |
| TNF-a | 0.68 (0.41-0.95) | 0.20 |
| hs-Troponin | 0.67 (0.44-0.89) | 0.15 |
| Soluble TIE2 | 0.67 (0.44-0.89) | 0.32 |
| Syndecan-1 | 0.65 (0.42-0.87) | 0.20 |
| IL-6 | 0.64 (0.38-0.90) | 0.30 |
| VEGF-D | 0.64 (0.40-0.88) | 0.27 |
| D_4-6µm_ | 0.64 (0.35-0.92) | 0.34 |
| Angpt-2 | 0.62 (0.36-0.87) | 0.37 |
| TF | 0.62 (0.37-0.86) | 0.35 |
| D-Dimer | 0.61 (0.31-0.91) | 0.48 |
| sFLT-1 | 0.57 (0.34-0.80) | 0.56 |
| Ferritin | 0.57 (0.27-0.87) | 0.66 |
| V_RBC_ | 0.55 (0.29-0.80) | 0.71 |
| Sex | 0.53 (0.25-0.80) | 0.86 |
| BMI | 0.46 (0.21-0.71) | 0.76 |
| HA | 0.45 (0.20-0.70) | 0.70 |
| Angpt-1 | 0.38 (0.08-0.67) | 0.40 |

Abbreviations:

ACE2 = shed ectodomain of angiotensin-converting enzyme 2 receptor; ADAMTS13 = a disintegrin and metalloprotease with thrombospondin type 1 motif, member 13; Angpt-1 = Angiopoietin-1; Angpt-2 = Angiopoietin-2; ARDS = Acute respiratory distress syndrome; AUC = Area under the curve; BMI = Body mass index; CCI score = Charlson Comorbidity Index; CI = Confidence Interval; CRP = C-reactive protein; D_4-6µm_ = Capillary density; hs-Troponin = high-sensitive Troponin, HA = Hyaluronic acid, IL-6 = Interleukin-6, PBR = Perfused boundary region, PCT = Procalcitonin, sFlt-1 = Soluble Fms-like thyrosinkinase-1, Soluble Tie2 = Angiopoietin-1 receptor, Soluble TM = Soluble thrombomodulin, TNF-a = Tumor necrosis factor a, VEGF-A = Vascular endothelial growth factor A, VEGF-D = Vascular endothelial growth factor D, V_RBC_ = Capillary red blood cell velocity
