# Supplementary material for: Microvascular dysfunction in COVID-19: the MYSTIC study
Source: Angiogenesis. 2020 Oct 14;24(1):145–57. doi: 10.1007/s10456-020-09753-7 (PMC7556767; doi:10.1007/s10456-020-09753-7)
Supplement: Supplementary file 3 — Electronic supplementary material 3 (DOCX 29 kb) [file 10456_2020_9753_MOESM3_ESM.docx]

**Microvascular dysfunction in COVID-19 – the MYSTIC study**

Alexandros Rovas^*^, Irina Osiaevi^*^, Konrad Buscher, Jan Sackarnd, Phil-Robin Tepasse, Manfred Fobker, Joachim Kühn, Stephan Braune, Ulrich Göbel, Gerold Thölking, Andreas Gröschel, Hermann Pavenstädt, Hans Vink, Philipp Kümpers

* contributed equally and are both considered first authors

**Corresponding author**:

Philipp Kümpers MD, Department of Medicine D, Division of General Internal Medicine, Nephrology, and Rheumatology, University Hospital Münster, Albert-Schweitzer-Campus 1, 48149 Münster, Germany

Email: philipp.kuempers@ukmuenster.de

Phone: 0049-251-83-47516

# Online Data Supplement

## Assessment of the sublingual microcirculation

The SDF camera (CapiScope HVCS, KK Technology, Honiton, UK) uses green light emitting stroboscopic diodes (540 nm) to detect the hemoglobin of passing red blood cells (RBCs). Using a 5x objective with a 0.2 numerical aperture, images were captured, providing a 325-fold magnification in 720 x 576 pixels at 23 frames per second (1-3). The GlycoCheck^TM^ software (Microvascular Health Solutions Inc., Alpine, UT, USA) allows video acquisition after predefined image quality criteria (motion, intensity, and focus) are fulfilled. Each complete measurement consists of at least ten 2-second videos (40 frames/video), containing a total of about 3000 vascular segments of 10 μm length each. All videos are deliberately obtained from different positions to counterbalance spatial heterogeneity of the sublingual microcirculation. The software automatically subjects the vascular segments obtained to a strict quality check. After marking and discarding invalid segments, the software obtains up to 840 radial intensity profiles for each valid vascular segment and, based on the RBC column width (RBCW), automatically groups vessels from 4 to 25 µm diameter in 22 separate diameter classes (1µm each). Data from two complete sequential measurements were averaged post-hoc to avoid sampling error and to counterbalance spatial heterogeneity of the sublingual microcirculation.

**Glycocalyx dimensions *in vivo***

The software calculates the dynamic lateral movement of RBCs into the permeable part of the eGC layer, expressed as the PBR (perfused boundary region, in µm). An impaired eGC permits a greater number of RBCs to penetrate deep into the endothelium, which is translated as an increase in the PBR value. The radial distribution of RBCs in each valid segment defines the median RBC width (RBCW), as well as the outer edge of the RBC-perfused vessel diameter (D_perf_). The PBR is defined as the distance between the RBCW and D_perf_ and is calculated using the following formula: (D_perf_ – RBCW)/2. The PBR has been identified as a robust and reliable estimate of glycocalyx damage. (1, 3-5)

1. Dane MJ, Khairoun M, Lee DH, van den Berg BM, Eskens BJ, Boels MG, van Teeffelen JW, Rops AL, van der Vlag J, van Zonneveld AJ, Reinders ME, Vink H, Rabelink TJ. Association of kidney function with changes in the endothelial surface layer. *Clin J Am Soc Nephrol* 2014; 9: 698-704.

2. Lee DH, Dane MJ, van den Berg BM, Boels MG, van Teeffelen JW, de Mutsert R, den Heijer M, Rosendaal FR, van der Vlag J, van Zonneveld AJ, Vink H, Rabelink TJ, group NEOs. Deeper penetration of erythrocytes into the endothelial glycocalyx is associated with impaired microvascular perfusion. *PLoS One* 2014; 9: e96477.

3. Rovas A, Lukasz AH, Vink H, Urban M, Sackarnd J, Pavenstadt H, Kumpers P. Bedside analysis of the sublingual microvascular glycocalyx in the emergency room and intensive care unit - the GlycoNurse study. *Scand J Trauma Resusc Emerg Med* 2018; 26: 16.

4. Drost CC, Rovas A, Kusche-Vihrog K, Van Slyke P, Kim H, Hoang VC, Maynes JT, Wennmann DO, Pavenstadt H, Linke W, Lukasz A, Hesse B, Kumpers P. Tie2 Activation Promotes Protection and Reconstitution of the Endothelial Glycocalyx in Human Sepsis. *Thromb Haemost* 2019; 119: 1827-1838.

5. Rovas A, Seidel LM, Vink H, Pohlkotter T, Pavenstadt H, Ertmer C, Hessler M, Kumpers P. Association of sublingual microcirculation parameters and endothelial glycocalyx dimensions in resuscitated sepsis. *Crit Care* 2019; 23: 260.
